# Supplementary material for: The effect of eviction moratoria on the transmission of SARS-CoV-2
Source: Nat Commun. 2021 Apr 15;12:2274. doi: 10.1038/s41467-021-22521-5 (PMC8050248; doi:10.1038/s41467-021-22521-5)
Supplement: Supplementary file 1 — Supplementary Information [file 41467_2021_22521_MOESM1_ESM.pdf]

Supplementary Information

The effect of eviction moratoria on the transmission of SARS-CoV-2

Anjalika Nande[1*], Justin Sheen[2*], Emma L Walters[3], Brennan Klein[4,5], Matteo Chinazzi[4,5], Andrei H Gheorghe[1], Ben Adlam[1], Julianna Shinnick[2], Maria Florencia Tejeda[2], Samuel V Scarpino[4], Alessandro Vespignani[4,5], Andrew J Greenlee[3], Daniel Schneider[3], Michael Z Levy[2†], Alison L Hill[1,6†]

*These authors contributed equally
†These authors jointly supervised this work, alhill@jhmi.edu, mzlevy@upenn.edu

[1]Program for Evolutionary Dynamics, Harvard University, Cambridge, MA, 02138
[2]Department of Biostatistics, Epidemiology and Informatics, University of Pennsylvania Perelman School of Medicine, Philadelphia, PA 19104
[3]Department of Urban and Regional Planning, University of Illinois at Urbana-Champaign, Champaign, IL 61820
[4]Network Science Institute, Northeastern University, Boston, MA, USA
[5]Laboratory for the Modeling of Biological and Socio-technical Systems, Northeastern University, Boston, USA
[6]Institute for Computational Medicine, Johns Hopkins University, Baltimore, MD 21218

Table of Contents

Supplementary Note **3**
Model details and parameters 3
Calculating the secondary attack rate 3
Estimating R0 for heterogeneous networks 4
Estimating eviction rates across the US during the COVID-19 pandemic 5
Cluster specific down weighting of external contacts during intervention 6
Typological analysis of Philadelphia zip codes 7
Using mobility data to determine contact patterns and quantify social distancing in Philadelphia 8
Using mobile device data to estimate relocation patterns in Philadelphia 10
Data Availability 11
Code Availability 11

Supplementary Figures **12**

Supplementary Tables **27**

Supplementary References **32**

Supplementary Note

Model details and parameters

We simulate the spread of SARS-CoV-2 using a modified version of the standard SEIR (Susceptible, Exposed, Infectious, Removed) model that allows for gamma-distributed durations of infection and infection spread over a network of contacts. We estimate the duration of the latent period (E stage, time before onset of infectiousness) as 4$\pm$4 days, based on an incubation period duration of 5$\pm$4 days [1,2] and assuming 1 day of pre-symptomatic transmission (similar to other models [3,4], and based on estimates of the portion of transmission that is pre-symptomatic [5] and the observation that viral load peaks at/before time of symptom onset [6–8]). Then, we assume a serial interval distribution of 7.5$\pm$5 days (measured in the absence of rapid case isolation or other controls) [1,9–11] and back out an infectious period duration of 7$\pm$4 (length of I_1 state). This value is also consistent with estimates of the duration of high-level viral shedding [6] and of the symptomatic phase of mild (non-hospitalized) infection [11–14]. These are the main parameters governing the relationship between the input value of R_0 (which we use to back calculate the β's) and the rate of early exponential growth rate observed in the simulation before any social distancing.

Although not the focus of this work, our model also offers the ability to track the progression to more serious clinical stages of infection as well as recovery (R) and death (D) [15]. Here we use these other infection stages (i.e. hospitalization, ICU stay) only to include realistic approximations for the distribution of the timing from symptom onset to death (~20$\pm$10 days, in agreement with [13,16,17]) and for the portion of infected individuals who may eventually die (~1%, in agreement with [18–21]). Tracking deaths help us to recreate the trajectories of the epidemic across different US metropolitan areas (Figures 3, S3-8): Since seroprevalence surveys across the US have suggested that cases are massively underreported, we calibrate the model to reproduce the epidemic size in terms of death counts and use the IFR to infer total cases.

In results that report “seroprevalence”, this was extracted from the model as the fraction of all currently living residents who were in the R stage, which is a rough surrogate. For results reporting “final epidemic size” or cumulative prevalence, we counted all individuals who had ever been in any stage of infection (i.e. E, I, R, or D).

Calculating the secondary attack rate

The secondary attack rate (SAR) is defined as the fraction of contacts of an infected individual who are infected directly by them over the course of their infectious period. For an individual with infectious period of length T, the SAR is:

$$SAR = 1 - e^{-\beta T} \quad (1)$$

For a population of individuals in whom the infectious period is gamma distributed with mean *T* and shape parameter *k*, as in our model, the population-average SAR is :

$$SAR = \frac{(\beta T/k + 1)^k - 1}{(\beta T/k + 1)^k} \quad (2)$$

Considering transmission within households, we use observed values of the household SAR and our parameters of the infectious period (*T, k*) to back out the transmission rate within households:

$$\beta_{HH} = \left(\frac{k}{T}\right)\left(\left(\frac{1}{1-SAR_{HH}}\right)^{\frac{1}{k}} - 1\right) \quad (3)$$

Estimating R_0 for heterogeneous networks

We divide contacts down into two types - household and external - and similarly, the overall R_0 can be decomposed into two components:

$$R_0 = R_{HH} + R_{EX} \quad (4)$$

For a fixed uniform random network where everyone has *n* contacts, R_0 can be approximated by:

$$R_0 = \beta T\,(n - 1) \quad (5)$$

where the $-\ 1$ refers to the fact that an individual cannot infect the contact who infected them.

However, for random networks with significant variance in the number of contacts per individual, this formula substantially underestimates R_0. Early in an epidemic, highly-connected individuals are more likely to be infected, and so R_0 is more accurately estimated as:

$$R_0 = \beta\, T\left(\frac{<x^2>}{<x>} - 1\right) = \beta\, T(\ < x >\,(1 + CV^2) - 1) = \beta\, T(\,n\,(1 + CV^2) - 1) = \beta T\,(\,n' - 1) \quad (6)$$

where n is the average degree, $CV$is the coefficient of variation, and n' is an “effective” degree that takes into account the heterogeneity [22,23].

The variation in household sizes is small, so we use Eq 5 for R_0^{HH}, but we since we allow a large variation in external degree to account for realistic heterogeneity in human contact patterns and the degree of superspreading seen for SARS-CoV-2, we use Eq 6 for R_0^{EX}. The combined R_0 is therefore:

$$R_0 = \beta T(w_{HH} n_{HH} + w_{EX} n'_{EX} - (w_{HH} f_{HH} + w_{EX} f_{EX})) \quad (7)$$

We define the weights of the household (external) layer $w_{HH}(w_{EX})$ such that $\beta_{HH} = \beta\, w_{HH}$ and $\beta_{EX} = \beta\, w_{EX}$. The average contacts in each layer are w_{HH} and w_{EX}, and the effective degree of the external layer (taking into account variance in connectivity) is $n'_{EX} = n_{EX}(1 + CV^2_{EX})$. Instead of the -1 term seen for R_0 values for single layer networks (Eqs 5, 6), the $-w_i f_i$ value takes into account the fraction of infections caused by a particular contact type, with the terms given by $f_{HH} = \frac{w_{HH} n_{HH}}{w_{HH} n_{HH} + w_{EX} n'_{EX}}$ and $f_{EX} = 1 - f_{HH}$.

We define the weight of household contacts to be unity ($w_{HH} = 1$), so that the model value of β represents the rate of transmission over household contacts ($\beta = \beta_{HH}$). Then we back out the weight of external contacts (w_{EX}) from literature reports of the increased rate of transmissivity in households relative to outside (i.e. from reports of w_{EX}/w_{HH}).

We can then use Eq 7 with fixed values of R_0, T, w_{HH}, w_{EX}, n_{HH}, and CV_{EX} to back out a value of n_{EX}.

$$n = \frac{1}{2}\left(\left(\frac{R_0}{\beta T} + w_{EX}\right) + \sqrt{\left(\frac{R_0}{\beta T} + w_{EX}\right)^2 + 4\, w_{HH} n_{HH}(w_{HH} - w_{EX})}\right)$$

$$n'_{EX} = (n - w_{HH} n_{HH})/w_{EX} \quad (8)$$

$$n_{EX} = n'_{EX}/(1 + CV^2_{EX})$$

Based on the desired n_{EX} and CV_{EX}, the parameters of a negative binomial distribution with parameters p (probability of success) and r (number of successes) are:

$$r = n_{EX}/(CV^2_{EX}\, n_{EX} - 1)$$

$$p = r/(r + n_{EX}) \quad (9)$$

Estimating eviction rates across the US during the COVID-19 pandemic

To estimate the range of possible eviction rates across U.S. cities, we used data from Eviction Lab [24] and from an analysis by Stout [25]. Eviction rates are often expressed as rates per rental household so we scale the eviction rate per all households according to the percent of renter households in an area. Historically, across U.S. cities baseline evictions rates vary from ~0.1%/month to ~1%/month (Supplementary Figure 2). However, high unemployment rates due to COVID-19 have already increased the potential eviction rate by creating a backlog of eviction filings throughout the country that could move forward quickly if current eviction moratoria were removed or struck down [26], and the continued high unemployment rates will only increase the eviction rate in metropolitan regions compared to their historical baselines.

To account for the uncertainty in the growth of eviction filings due to COVID-19 over the next year, we look at the fold-increase in unemployment in the same cities (compared to 2019) and assume that eviction rates (without any policies preventing evictions) could be increased by the same amount. Unemployment data was from the Bureau of Labor Statistics via the Department of Numbers (Supplementary Figure 2A). In addition, we took estimates produced at a state-level by the consulting firm Stout, which used more detailed data on household income, savings, rent costs, unemployment, and recent national surveys (Supplementary Figure 2B). These analyses suggest that eviction rates up to ~2%/month are reasonable, though even higher rates are estimated with this method for some regions of the country. We consider the following eviction rates: 0% (comparison case), 0.1%, 0.25%, 0.5%, 1%, 2%/month which represent the spectrum of eviction rates for the majority of metropolitan regions.

Cluster specific down weighting of external contacts during intervention

Two clusters: In the case where the population was divided into two clusters - one for high socioeconomic status (SES) households and one for low SES households, there are three types of external connections that can occur. Connections between individuals belonging to high SES households (x_{11}), those between low SES households (x_{22}) and connections between one individual in a high SES household with one in a low SES household (x_{12}). We reduce the weights of each type of contact ($r_{11}, r_{22}, r_{12} < 1$) such that the effective intervention efficacy of each cluster is reduced by the desired amounts ($\eta_1, \eta_2 < 1$). The relationship between the reduction in weights of each type of contact and the effective intervention efficacy of each cluster can be calculated via the following system of equations,

$$r_{11}x_{11} + r_{12}0.5\,x_{12} = \eta_1(x_{11} + 0.5\,x_{12})$$

$$r_{12}\,0.5x_{12} + r_{22}x_{22} = \eta_2(0.5x_{12} + x_{22})$$

This system of equations is underdetermined as it has two equations and three unknowns (r_{11}, r_{22}, r_{12}). We fix $r_{12} = r_{22} = \eta_2$ and solve for $r_{11} = \frac{\eta_1(x_{11} + 0.5x_{12}) - \eta_2\,0.5x_{12}}{x_{11}}$.

Typological analysis of Philadelphia zip codes

Socio-economic indicators relevant to describing housing stability and COVID vulnerability were used to perform principal component analysis (PCA) and clustering to classify neighborhood types in the city of Philadelphia [27]. Data was collected by zip code tabulation areas from the 2019 US Census. The full list of indicators used and their value for each cluster is provided in Supplementary Table 3.

PCA and clustering resulted in three typologies (shown in Figure 5A): a higher income rental neighborhood, a moderate income and working class owner neighborhood, and a low income rental neighborhood. Cluster 1, the higher income neighborhood, is defined by high incomes and high housing costs. Cluster 2 is a slightly older, ownership centered neighborhood with lower poverty rates. Cluster 3, the lower income rental neighborhood, is defined by higher poverty rates, lower incomes, more children, and higher rates of service industry employment and essential workers. Details of these clusters are provided below. Considering the socio-economic stratification among these typologies, it's likely that Type 3 zip codes will face higher levels of health and socio-economic vulnerabilities due to COVID-19. We then analyzed the population, household, and rental household tabulations, race, ethnicity, and eviction rates of each cluster, which were variables not included during the PCA.

To get the monthly eviction rate per household in Philadelphia for each cluster, we use eviction rate data reported for each zip code, from Eviction Lab [24]. Eviction Lab reports eviction rate per rental unit per year, and we used the reported fraction of all households that were renters (vs owners) to reframe the eviction rate as per all households per month.

<u>Cluster 1:</u> Cluster 1 is defined by higher housing costs and higher income when compared to the overall mean of the city. When compared to the overall mean, these zip codes have significantly higher median home values, higher gross rents, higher incomes, and a higher per capita income. They are predominantly rental units with lower rates of poverty and cost burdened households than the overall mean. Additionally, the percentage of residents who are essential workers is lower than the overall mean of the city. Considering the high housing costs, lower cost burdened rates, and lower rates of essential workers, it's likely that these zip codes are economically stable and less vulnerable to the health and socio-economic impacts of COVID-19. When analyzing race and ethnicity of the clusters (see Supplementary Table 3), Cluster 1 zip codes are predominantly white with a small share of the population being residents

of color. Cluster 1 zip codes also see the lowest eviction filing rates and eviction rates at 3.3% and 1.45% per rental household per year, respectively.

Cluster 2: Cluster 2 is defined by higher rates of homeownership and a slightly older population compared to the overall mean. Vacancy rates, poverty rates, and mobility rates are lower than the overall mean. When analyzing the overall means of all variables in this cluster (see Supplementary Table 1), the median household income is about equal to the overall mean and the median home value is just above the overall mean. Additionally, these zip codes have higher rates of essential workers among their residents when compared to the city's overall mean. Considering the socio-economic conditions of this cluster, it's likely that this type of neighborhood is moderate income and working class. Cluster 2 neighborhoods are far more diverse than cluster 1 neighborhoods (Supplementary Table 3), but still skew white with the overall mean of the white population in zip codes in cluster 1 being 51 percent. Cluster 2 sees a higher concentration of Black residents and a slight increase in the Latino population when compared to cluster 1. Zip codes in cluster 1 also see substantially higher eviction filing rates and eviction rates compared to cluster 1. The mean eviction filing rate and eviction rate are 7.8% and 3.8% per rental household per year, respectively.

Cluster 3: Cluster 3 represents lower income zip codes with higher rates of female headed households, higher poverty rates, higher vacancy rates, lower home values, and lower incomes when compared to the overall means of these indicators. These zip codes also have higher rates of residents employed in the service industry and as essential workers. This indicates that households in this type of neighborhood are more vulnerable to the health and socio-economic impacts of COVID-19. Zip codes in cluster 3 are predominantly nonwhite with higher Black and Latino populations than cluster 1 and 2. Eviction filing rates and evictions rates are also higher in cluster 3 zip codes than in other areas of the city with an eviction filing rate of 10.2% and an eviction rate of 4.7% per rental household per year.

Using mobility data to determine contact patterns and quantify social distancing in Philadelphia

Throughout the COVID-19 pandemic, aggregated mobile device data has been used for building and parameterizing epidemic models [28–32], as well as studying the impact of the pandemic on our mobility and social contacts [33–37]. Here, we use aggregated mobile phone data from more than 13,000 opted-in, anonymous users in Philadelphia, PA, USA to estimate the probability that residents of different zip codes (or zip code clusters) interact with one another in a given day based on co-location. This results in an estimated contact matrix where each entry corresponds to the average number of interactions that a resident of zip code z_i has with residents of zip code z_j. We use data from Cuebiq, which provides data to academic and humanitarian initiatives through its Data for Good program (https://www.cuebiq.com/about/data-for-good/). These data are first-party and collected from anonymous users who have opted in to share their location data. The privacy of these users is further enhanced in several ways: First, users' "personal

areas" such as home locations are up-leveled to the Census block group level, which preserves key demographic properties while obscuring sensitive location information. Second, the measures derived from these data are again aggregated to the zip code level, further eliminating any identifiable information of the users. We have used these data with similar processing schemes in previous COVID-19-related papers [28,36,38,39].

Selecting a panel of users

In this study we consider mobility data from 13,333 users that have personal areas in the city of Philadelphia. These users were sampled from a larger panel of more than 5 million users nationwide. This original panel represents a subset of all active users in the Cuebiq dataset who met the following criteria: 1) users who were active in the dataset for at least 21 days during each month from January to June 2020, 2) users whose devices reported on average at least one location ping per hour, and 3) users with an average device geolocation accuracy of less than 50 meters.For more details about the criteria and composition of the panel, as well as the rationale for the selection criteria, see Klein et al. [36]. To see an interactive dashboard of different collective physical distancing measures that were computed with these data, see http://covid19.gleamproject.org/mobility.

Operationalizing opportunities for contact in mobile device data

We define an *opportunity for contact* as two devices being spatially co-located for some period of time. Here, spatial co-location is based on the longitude-latitude position of two devices, such that if two devices are within the same 8-character *geohash*, they qualify as being co-located. A geohash is a compressed string representation of the full longitude-latitude coordinates, and the more characters in a geohash, the finer scale resolution it will have; 8-character geohash are typically 25m^2, with the largest dimensions being 19m x 38m at the equator [40]. Following guidance from the CDC about what constitutes a close contact, we treat co-location events of 15 minutes or more as a successful opportunity for contact. In sum: we define an opportunity for contact as a pair of devices being within the same 8-character geohash for 15-minutes or more.

Spatially-aggregated contact patterns across zip codes

In order to arrive at an aggregated cluster-to-cluster contact probability matrix, we first estimate a zip code-to-zip code contact matrix. This is done by first assigning each user to a "personal area" (i.e., an inferred home location—up-leveled to the zip code to preserve privacy—based on periods of nightly inactivity). Using these personal areas, we can estimate the likelihood that a resident of zip code z_i interacts with a resident of a nearby zip code z_j based on the sum of observed contact opportunities between users with personal areas of z_i and z_j.

We re-weight observed contacts between users with personal areas in zip codes z_i and z_j in order to account for possible sampling bias in our panel of users. To do this, we first define f_i to be n_i / N_i, which is the number of users with personal areas in the zip code, n_i, divided by the total population of the zip code, N_i. We observed a high correlation between n_i and N_i (e.g. a relatively constant value of across f_i zip codes, Supplementary Figure 16), and when we inferred demographic properties of (anonymous) Cuebiq users from their zip codes the distribution generally matched that of the city as a whole (Supplementary Table 7), highlighting the representative nature of this data. The users in the Cuebiq data were slightly more likely to be from zip codes with higher-than-average proportions of residents who were white, who were over 65, and who were homeowners. The adjusted estimate for the total number of contacts between a pair of zip codes z_i and z_j is defined as $X_{ij} = c_{ij} / (f_i * f_j)$, where c_{ij} is the observed number of contacts between users with personal areas in zip codes z_i and z_j. We normalize this to a per capita level within a given zip code by dividing by the population of that zip code, $x_{ij} = X_{ij} / N_i$. When referring to "contacts" between a pair of regions (e.g. zip codes or clusters), we are referring to this quantity.

At the zip code level, we are now left with a 46 x 46 weighted contact matrix, $\mathbf{X}_\tau$, where τ denotes a time frame of interest. We define $\mathbf{X}_{\mathbf{ref}}$ to be the baseline contact matrix, corresponding to the average activity between January 16 and February 28, 2020, excluding holidays (as in Klein et al. [36]). Each week's average contact patterns between pairs of zip codes can now be compared to the "typical" or baseline period, which was chosen because it represents activity after winter holidays and before large scale mobility disruptions were observed nationwide. Together, this lets us define the final matrix of interest, which describes the *percent of typical contacts between regions*. That is, elements W_{ij} of the matrix $\mathbf{W}_\tau = \mathbf{X}_\tau/\mathbf{X}_{\mathbf{ref}}$ correspond to the percent of typical activity between zip codes z_i and z_j. Lastly, we aggregate this into a cluster-to-cluster (3 x 3) matrix by taking the weighted sum of the elements of $\mathbf{W}_\tau$ (zip code to zip code contacts); for every zip code in a given cluster, we add together the average contacts proportionally based on the typical total number of contacts, $x_i = \sum x_{ij}$.

Note that we exclude zip code 19112 from our analyses for privacy reasons due to its small population (estimates on the order of n=10 residents according to the 2019 ACS Survey).

Using mobile device data to estimate relocation patterns in Philadelphia

As mentioned above, we assume that users are more likely to relocate within the same cluster in the event of an eviction. We validate that assumption by studying the change in personal areas in our panel of users. In order to calculate this percentage, we recompute the (up-leveled) average nighttime location of the users in our panel during February and October, 2020. We use this position as a coarse estimate of the home zip code of these users. Among users whose home zip codes changed between February and October, we construct a matrix corresponding

to the percent of relocations to each of the other zip codes in Philadelphia. In order to ensure user privacy, we only report percentages in this analysis.

The results of this analysis are shown in Supplementary Figure 17, where each element of this "relocation matrix" represents the percent of users who started (in February) with a home zip code in a given cluster and ended up (in October) with a home zip code in another cluster. Note that the large majority of relocations take place within the same cluster. Users in Cluster 1 are more likely to relocate to other clusters than those in Cluster 2 and Cluster 3. While more systematic demographic analyses of these within-city relocation patterns are surely warranted, we use the results in Supplementary Figure 17 only to parameterize our estimates of the rate of within- and between-cluster relocations for the epidemic simulations studied here.

Data Availability

All COVID-19 case and death data used in this study were downloaded from the publically-available New York Times repository in Github:
https://github.com/nytimes/covid-19-data

Data on the distribution of US household sizes and on socio-demographic indicators of zipcodes in Philadelphia was obtained from the 2019 United States Census [41]:
https://www.census.gov/data/tables/2019/demo/families/cps-2019.html

Mobility data used in this study is available from Cuebiq through their Data for Good program (https://www.cuebiq.com/about/data-for-good/). Restrictions apply to the availability of this data, which was used under license for the current study, and so are not publically available. All data aggregated at the level of zip-code clusters that was used in the models is presented in the Supplementary Tables. More detailed data is available from the authors upon reasonable request and permission from Cuebiq, and any researchers interested in working with the data can apply for an independent license from Cuebiq. Aggregated mobility metrics at the national, state, and CBSA level are publically available at https://covid19.gleamproject.org/mobility

Code Availability

All our simulation code is available in a Github repository [42]:
https://github.com/alsnhll/COVID19EvictionSimulations. The repository also contains code for downloading and processing COVID-19 case and death data from the New York Times repository.

Supplementary Figures

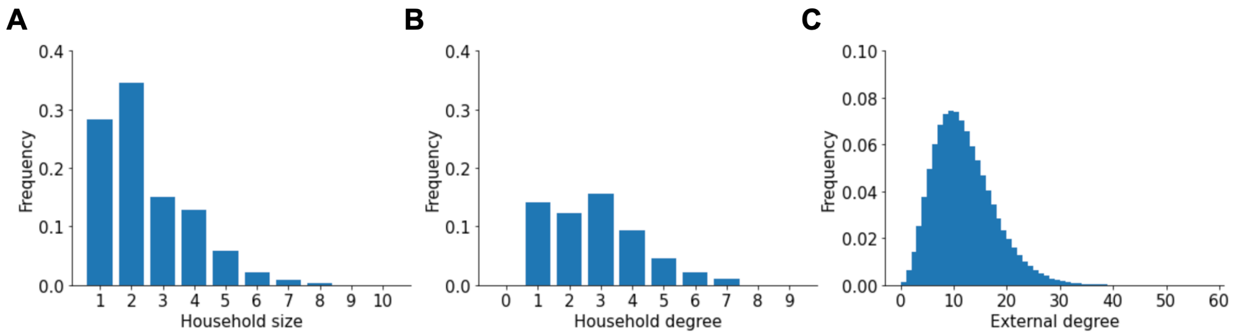

Supplementary Figure 1: Degree distributions in the network. A) Distribution of household sizes. B) Distribution of the number of household contacts (degree). C) Distribution of the number of non-household (external) contacts.

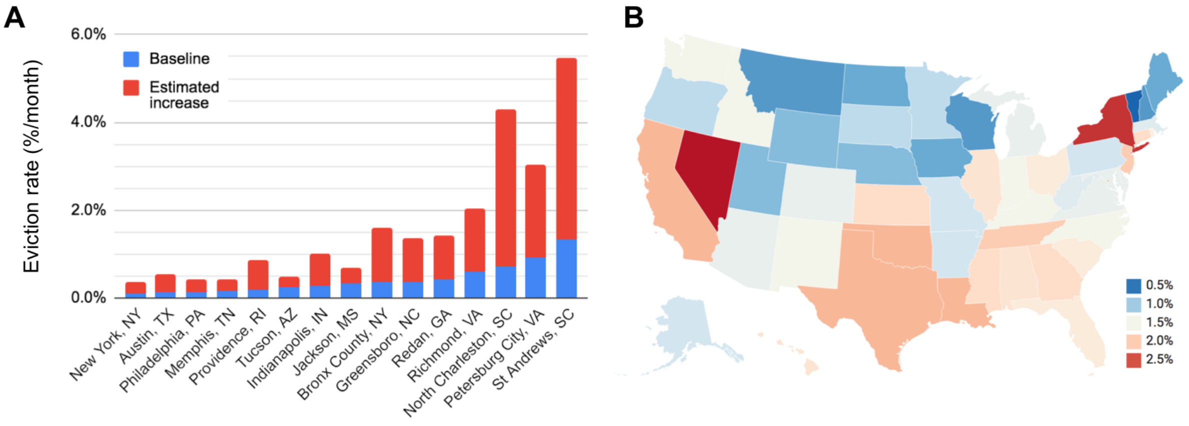

Supplementary Figure 2: Baseline and estimated future eviction rates across selected US cities/districts and states. A) Baseline eviction rates (blue) and estimated increase in eviction rate based on increased unemployment rate (red). Data from Eviction Lab [24]. Rates are percent of all households experiencing eviction per month. In a city of ~1 million people and a US-average household size of ~2.5, an eviction rate of 0.1%/month corresponds to about 400 evictions per month, and a rate of 2%/month works out to 8000/month. These cities were chosen to represent diversity in size, geography, and eviction rates B) Estimates for the percent of households facing eviction per month over the next four month in each state. Values came from an analysis from consulting firm Stout [25] and were based on US census data and surveys.

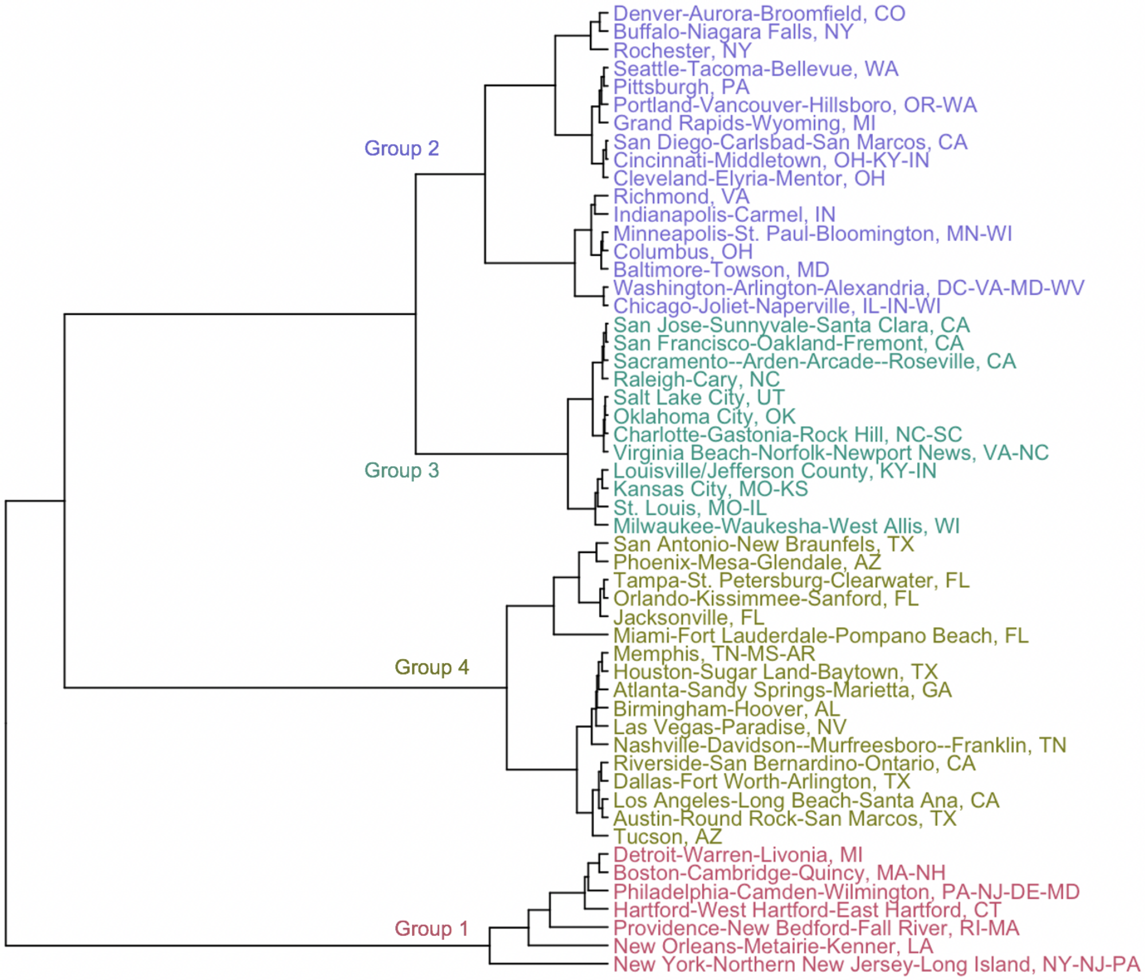

Supplementary Figure 3: Clustering cities based on COVID-19 epidemic trajectories. Dendrogram from results of hierarchical clustering of US metropolitan statistical areas based on the trajectory of COVID-19 cases and deaths through Sept 1 2020. City names are colored based on group membership when four clusters were chosen. See Methods for details.

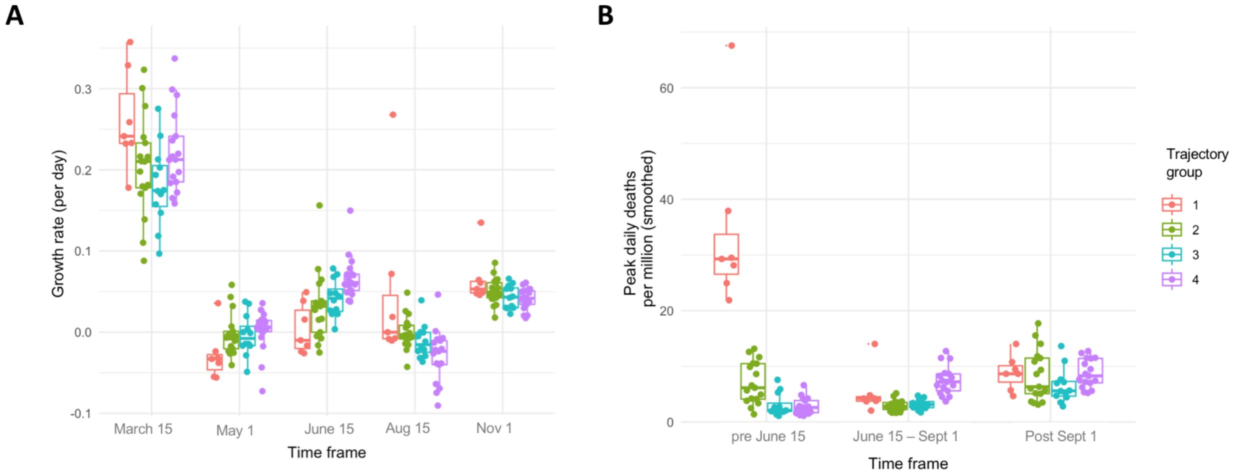

Supplementary Figure 4: Characteristics of the epidemic trajectory in each group of cities. A) Growth rates cases, measured for two weeks starting from the stated date. B) Peak deaths observed in each stated date interval. Box plots show median as line within box and IQR as bounds of the box. Whisker tips are the more extreme of either 1.5xIQR from box edge or maximum (minimum) data point. . All metrics were extracted from rolling 7-day averages of the daily incidence of new cases or deaths. See Methods for details.

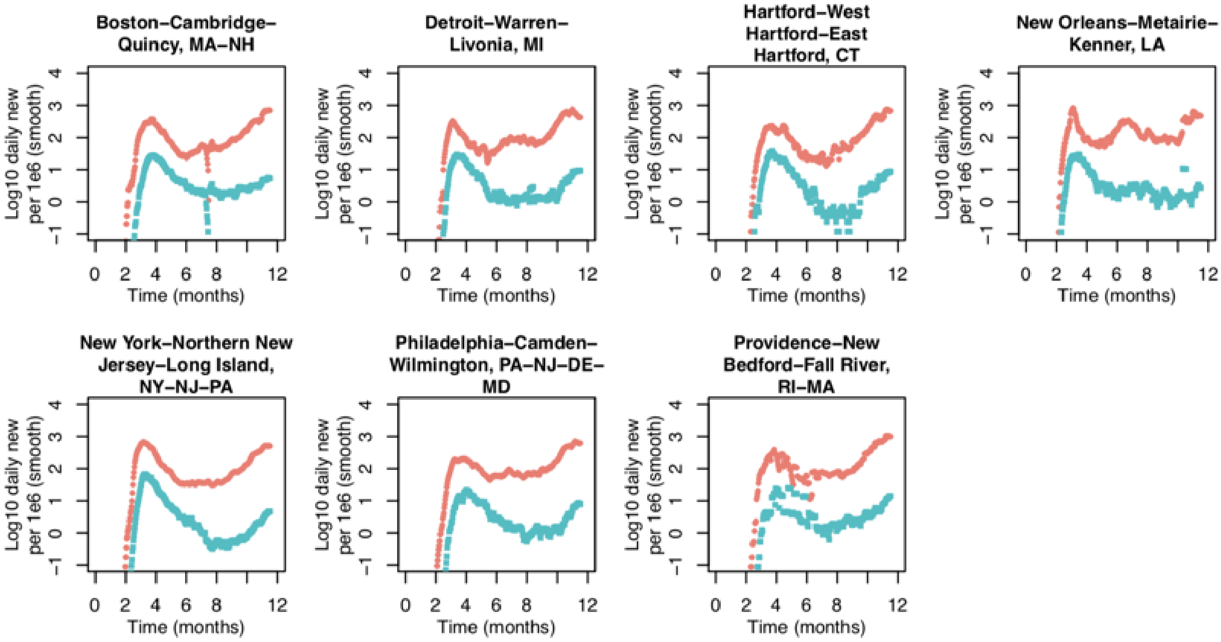

Supplementary Figure 5: Cities following "Trajectory 1" of COVID-19 cases and deaths. Daily incidence of cases and deaths (per million, 7-day rolling average) in US metropolitan statistical areas that were assigned to the "Trajectory 1" group by hierarchical clustering of the time series. Note that the anomalously low cases in mid August in the Boston area are due to an approximately one week lapse in reporting at the county level in Massachusetts. The irregularities in the Providence area data are due to irregular county-level reporting in Rhode Island.

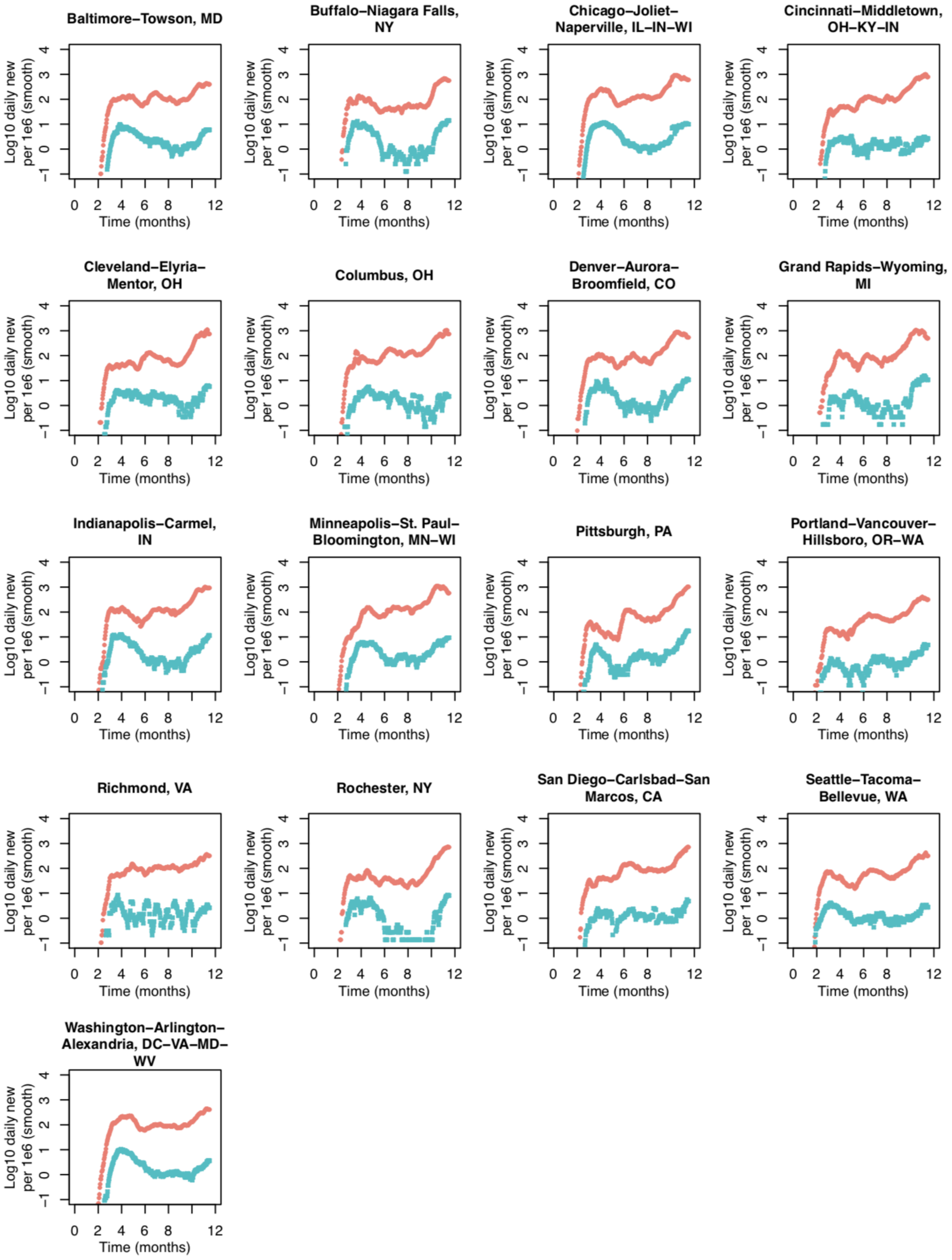

Supplementary Figure 6: Cities following "Trajectory 2" of COVID-19 cases and deaths. Daily incidence of cases and deaths (per million, 7-day rolling average) in US metropolitan statistical areas that were assigned to the "Trajectory 2" group by hierarchical clustering of the time series.

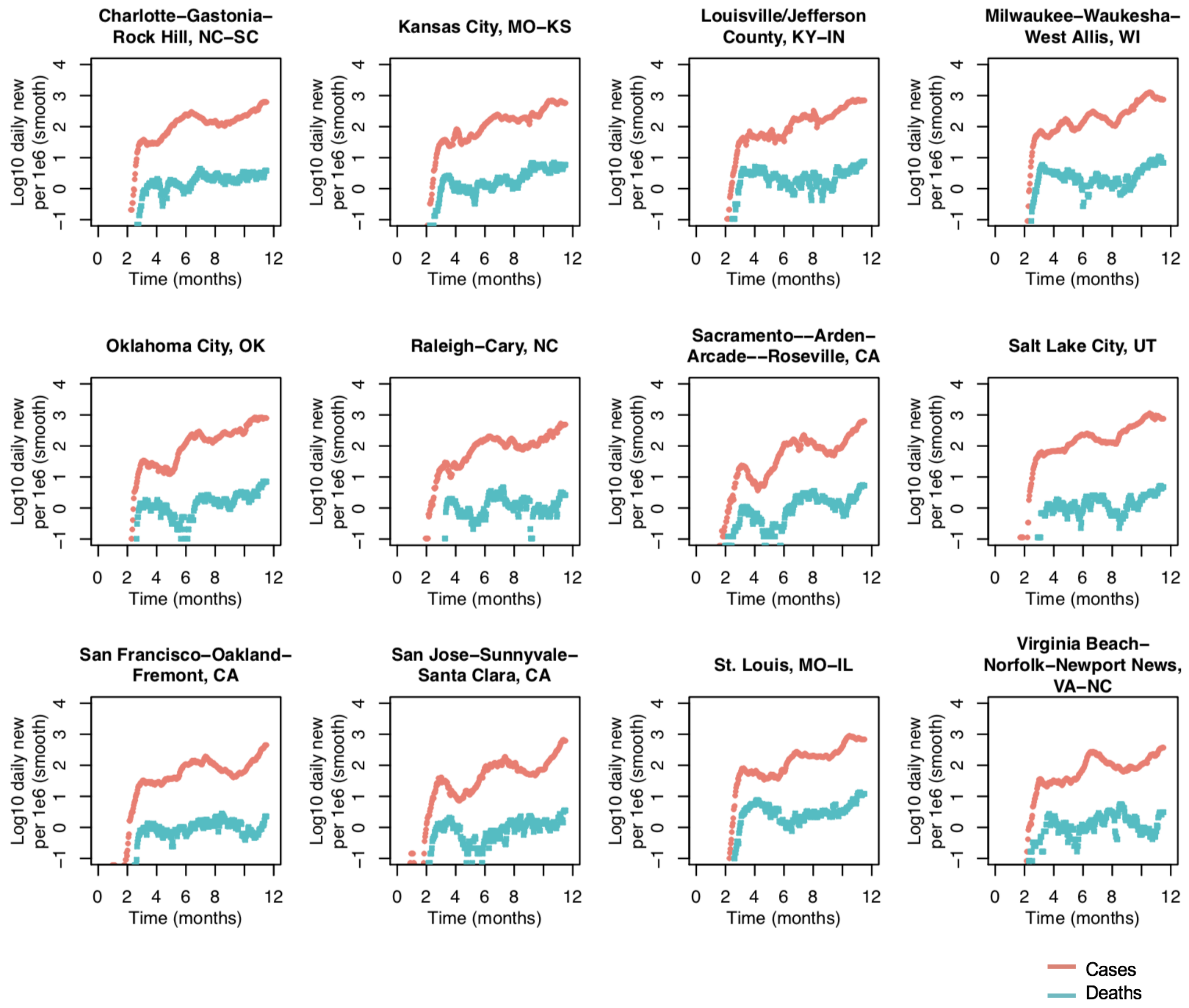

Supplementary Figure 7: Cities following "Trajectory 3" of COVID-19 cases and deaths. Daily incidence of cases and deaths (per million, 7-day rolling average) in US metropolitan statistical areas that were assigned to the "Trajectory 3" group by hierarchical clustering of the time series.

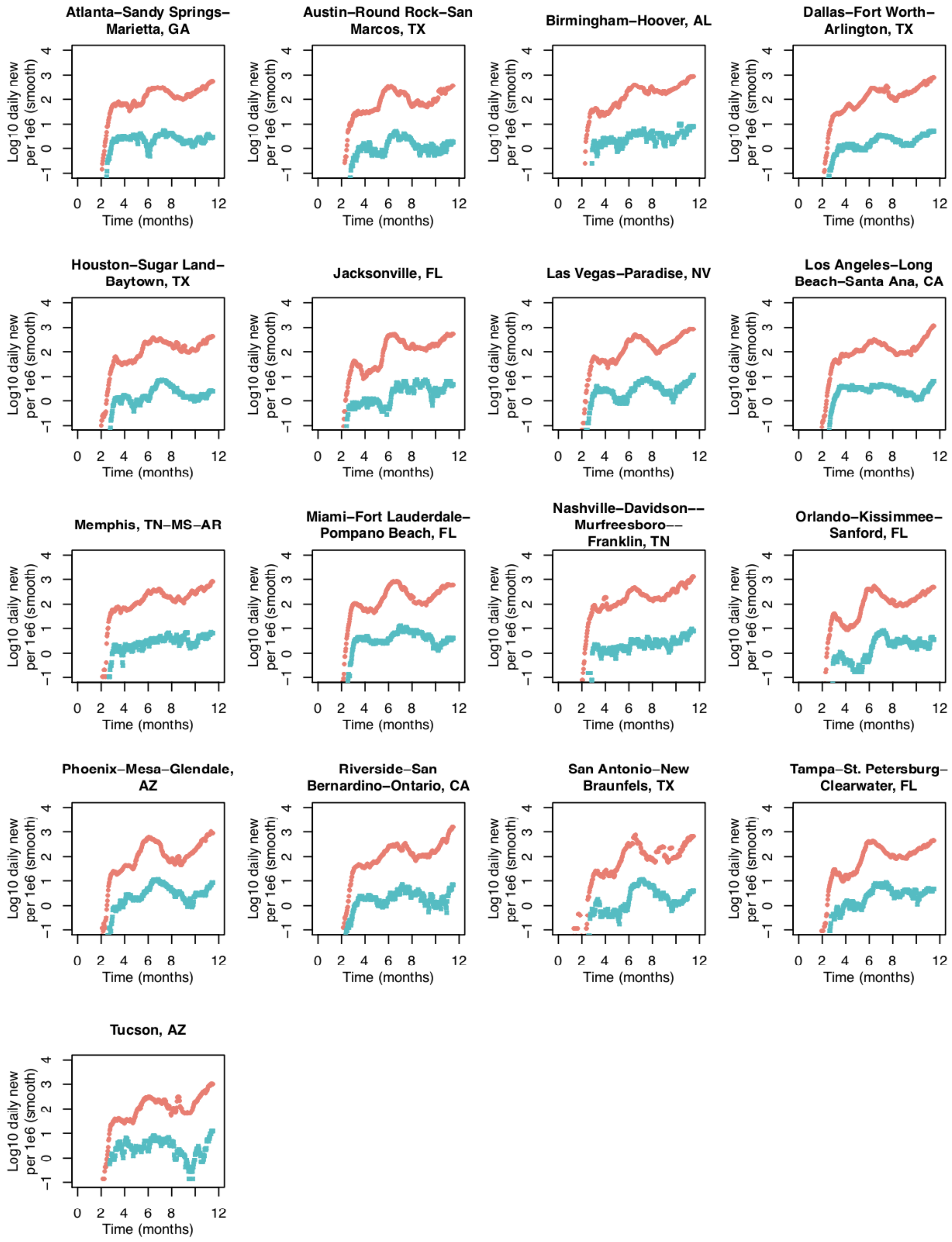

Supplementary Figure 8: Cities following “Trajectory 4” of COVID-19 cases and deaths. Daily incidence of cases and deaths (per million, 7-day rolling average) in US metropolitan statistical areas that were assigned to the “Trajectory 4” group by hierarchical clustering of the time series.

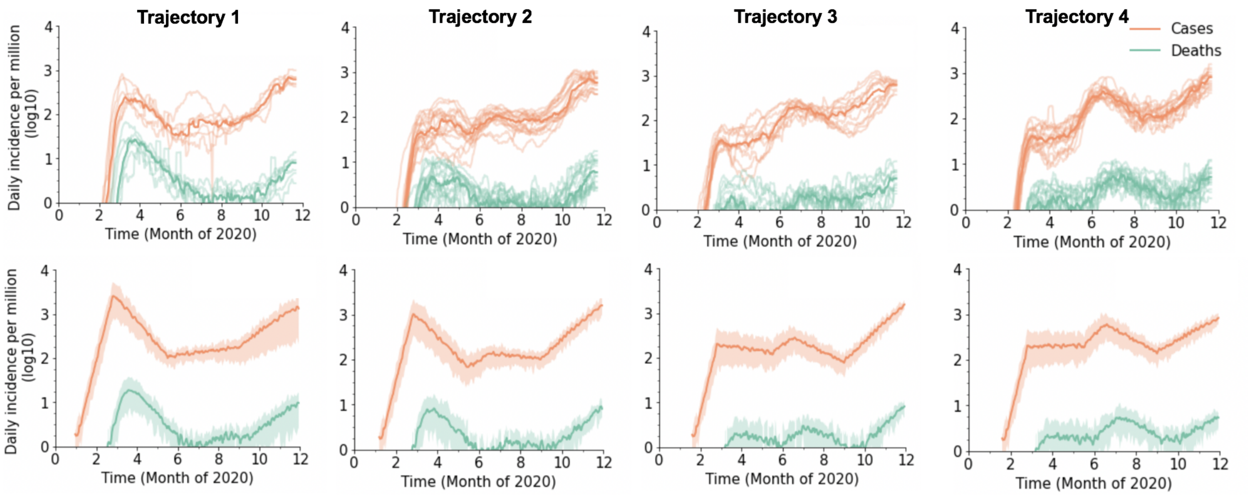

Supplementary Figure 9: Calibrated model trajectories for each group of cities. All panels show daily incidence of new cases and deaths per million individuals, applying a 7-day rolling average. Top row: Case and death data for each metropolitan area assigned to each trajectory group by the clustering algorithm (see Supplementary Figure 3). along with the group median (bolded line). Bottom row: Simulated case and death values from model scenarios calibrated to each group; shaded regions represent central 90% of all simulations. Each simulated trajectory consists of up to five distinct phases of the epidemic wherein the number of external contacts is reduced by a fixed amount. The phases are the early exponential phase (pre March 25), the spring period of control/lockdown (March 25 - June 15), a relaxation of controls and possible summer resurgence (June 15 - July 15), an optional re-imposition of some controls (July 15 - Oct 1), and a fall comeback (Oct 1 onwards). Parameters describing each trajectory are given in Supplementary Table 1. Note that in the data, cases are likely under-reported, especially during early phases of the epidemic, whereas the simulation counts all infections, so produces a higher ratio of cases to deaths.

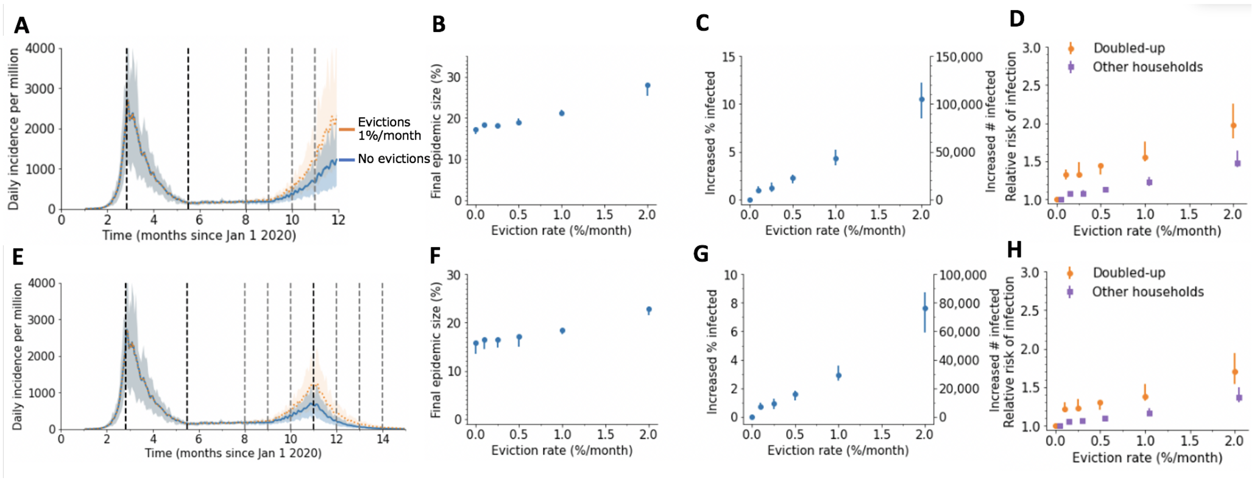

Supplementary Figure 10: Impact of evictions on a SARS-CoV-2 comeback during Fall 2020 for a higher household SAR. We model evictions occurring in the context of an epidemic similar to cities following “Trajectory 1”, with a large first wave and control in the spring, followed by relaxation to a plateau over the summer and an eventual comeback in the fall. Monthly evictions start Sept 1, with a 4-month backlog processed on the first month. The household secondary attack rate (SAR) was 0.5. A) The projected daily incidence of new infections (7-day running average) with and without evictions. Shaded regions represent central 90% of all simulations.The first lockdown (dotted vertical line) reduced external contacts by 85%, under relaxation (second dotted line) they were still reduced by 70%, and during the fall comeback they were reduced by 60% (fourth dotted line). B) Final epidemic size by Dec 31 2020, measured as percent of individuals who had ever been in any stage of infection. C) The predicted increase in infections due to evictions through Dec 31 2020, measured as excess percent of population infected (left Y-axis) or number of excess infections (right Y-axis). D) Relative risk of infection in the presence versus absence of evictions, for individuals who merged households due to evictions (“Doubled-up”) and for individuals who kept their pre-epidemic household (“Other households”). Data in B)-D) shown as median values with interquartile ranges across simulations. E)-H) Same as above but assuming a second lockdown is instituted on Dec 1, and maintained through March 2021.

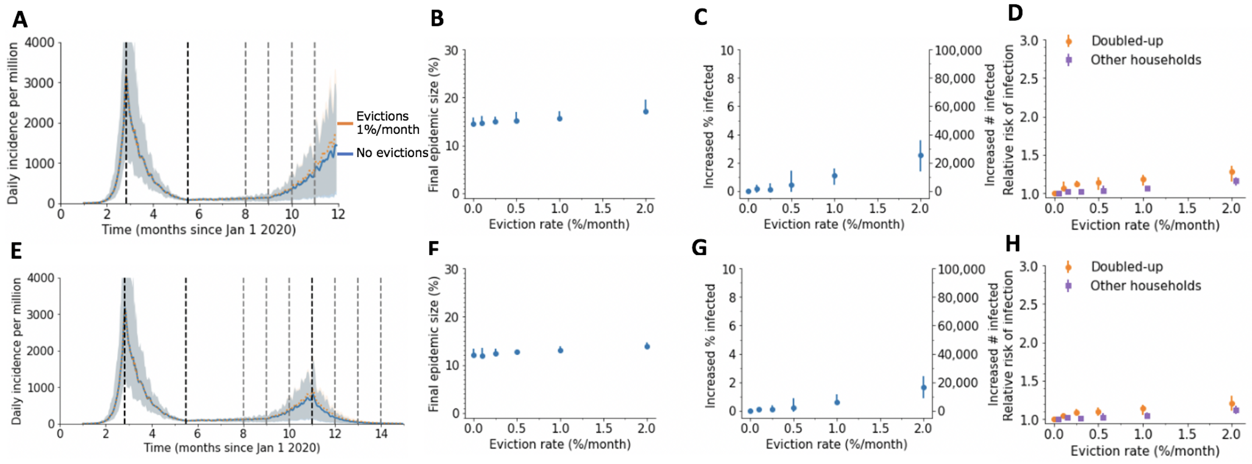

Supplementary Figure 11: Impact of evictions on a SARS-CoV-2 comeback during Fall 2020 for a lower household SAR. We model evictions occurring in the context of an epidemic similar to cities following "Trajectory 1", with a large first wave and control in the spring, followed by relaxation to a plateau over the summer and an eventual comeback in the fall. Monthly evictions start Sept 1, with a 4-month backlog processed on the first month. The household secondary attack rate (SAR) was 0.1. A) The projected daily incidence of new infections (7-day running average) with and without evictions. Shaded regions represent central 90% of all simulations.The first lockdown (dotted vertical line) reduced external contacts by 85%, under relaxation (second dotted line) they were still reduced by 70%, and during the fall comeback they were reduced by 60% (fourth dotted line). B) Final epidemic size by Dec 31 2020, measured as percent of individuals who had ever been in any stage of infection. C) The predicted increase in infections due to evictions through Dec 31 2020, measured as excess percent of population infected (left Y-axis) or number of excess infections (right Y-axis). D) Relative risk of infection in the presence versus absence of evictions, for individuals who merged households due to evictions ("Doubled-up") and for individuals who kept their pre-epidemic household ("Other households"). Data in B)-D) shown as median values with interquartile ranges across simulations. E)-H) Same as above but assuming a second lockdown is instituted on Dec 1, and maintained through March 2021.

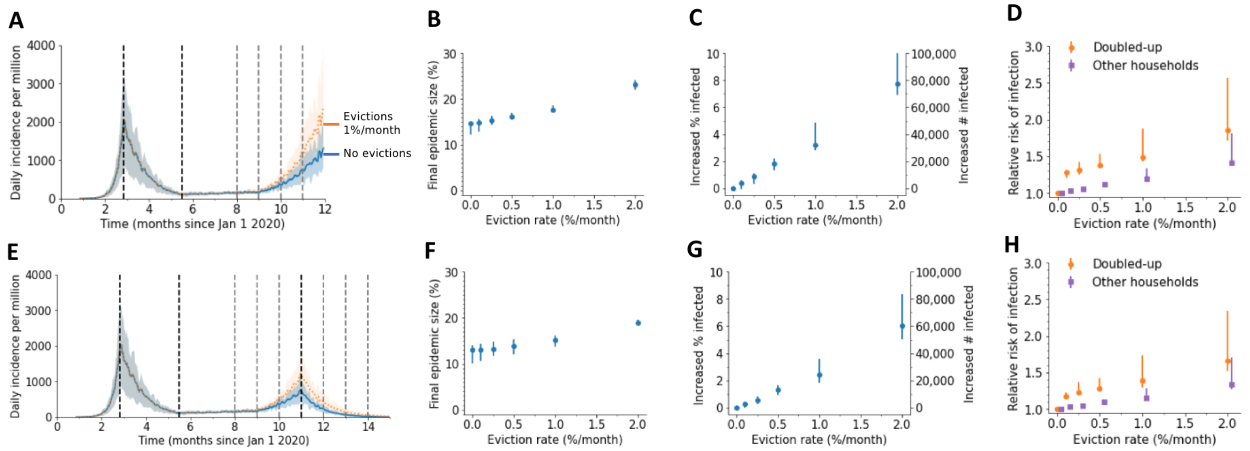

Supplementary Figure 12: Impact of evictions on a SARS-CoV-2 comeback during Fall 2020 when household and external contacts have equal transmission risk. We model evictions occurring in the context of an epidemic similar to cities following “Trajectory 1”, with a large first wave and control in the spring, followed by relaxation to a plateau over the summer and an eventual comeback in the fall. Monthly evictions start Sept 1, with a 4-month backlog processed on the first month. A) The projected daily incidence of new infections (7-day running average) with and without evictions. Shaded regions represent central 90% of all simulations.The first lockdown (dotted vertical line) reduced external contacts by 85%, under relaxation (second dotted line) they were still reduced by 70%, and during the fall comeback they were reduced by 60% (fourth dotted line). B) Final epidemic size by Dec 31 2020, measured as percent of individuals who had ever been in any stage of infection. C) The predicted increase in infections due to evictions through Dec 31 2020, measured as excess percent of population infected (left Y-axis) or number of excess infections (right Y-axis).D) Relative risk of infection in the presence versus absence of evictions, for individuals who merged households due to evictions (“Doubled-up”) and for individuals who kept their pre-epidemic household (“Other households”). Data in B)-D) shown as median values with interquartile ranges across simulations. E)-H) Same as above but assuming a second lockdown is instituted on Dec 1, and maintained through March 2021.

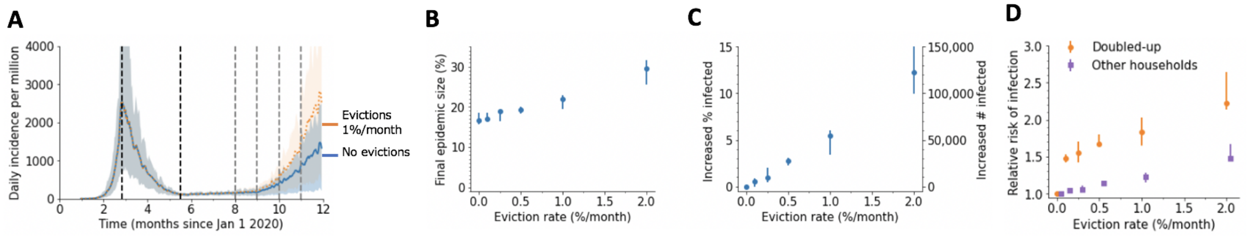

Supplementary Figure 13: Impact of evictions on a SARS-CoV-2 comeback during Fall 2020 when doubling up occurs with a connected household. We model evictions occurring in the context of an epidemic similar to cities following “Trajectory 1”, with a large first wave and control in the spring, followed by relaxation to a plateau over the summer and an eventual comeback in the fall. Monthly evictions start Sept 1, with a 4-month backlog processed on the first month. A) The projected daily incidence of new infections (7-day running average) with and without evictions. Shaded regions represent central 90% of all simulations.The first lockdown (dotted vertical line) reduced external contacts by 85%, under relaxation (second dotted line) they were still reduced by 70%, and during the fall comeback they were reduced by 60% (fourth dotted line). B) Final epidemic size by Dec 31 2020, measured as percent of individuals who had ever been in any stage of infection. C) The predicted increase in infections due to evictions through Dec 31 2020, measured as excess percent of population infected (left Y-axis) or number of excess infections (right Y-axis). D) Relative risk of infection in the presence versus absence of evictions, for individuals who merged households due to evictions (“Doubled-up”) and for individuals who kept their pre-epidemic household (“Other households”). Data in B)-D) shown as median values with interquartile ranges across simulations. Forcing doubling-up to occur with a household that at least one member of the evicted household had an existing direct external connection with, as opposed to with a random household, has two effects. Firstly, it reduces by one the number of new external connections gained by a household after doubling up. Secondly, it means that the households chosen to double-up with now have slightly higher-than-average connectivity (since the likelihood of choosing it increases with the number of external connections). Overall, the second effect dominates and in this scenario the impact of evictions is higher compared to the scenario where doubling-up is with a random household.

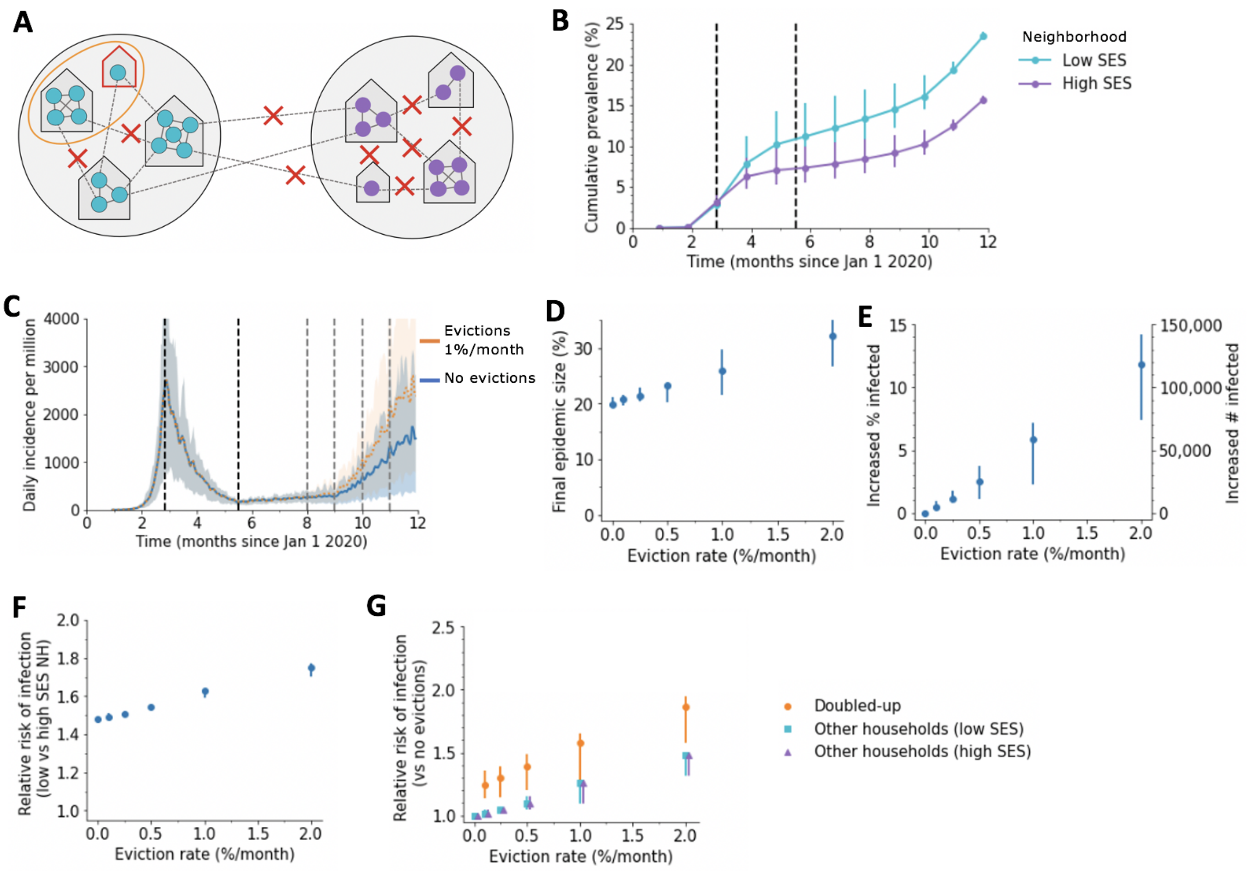

Supplementary Figure 14: Impact of evictions on COVID-19 epidemics in heterogeneous cities with preferential mixing. A) Schematic of our model for inequalities within a city. The city is divided into a "high socioeconomic status (SES)" (purple) and a "low SES" (teal) neighborhood. Evictions only occur in the low SES area, and individuals living in this area are assumed to be less able to adopt social distancing measures, and hence have higher contact rates under interventions (90% vs 80% reduction in external contacts during lockdown for 85% overall, 75% vs 65% during relaxation for 70% overall, and 65% vs 55% during fall comeback for 60%). Before interventions, residents are more likely (75% external contacts are within one's neighborhood) to contact someone outside the household who lives within vs outside their neighborhood. B) Cumulative percent of the population infected over time, by neighborhood, in the absence of evictions. C) The projected daily incidence of new infections (7-day running average) with 1%/month evictions vs no evictions. Shaded regions represent central 90% of all simulations. D) Final epidemic size by Dec 31 2020, measured as percent individuals who had ever been in any stage of infection, for the heterogenous city as compared to a homogenous city with same effective eviction rate and intervention efficacy. E) The predicted increase in infections due to evictions through Dec 31 2020, measured as excess percent of population infected (left Y-axis) or number of excess infections (right Y-axis). F) Relative risk of infection by Dec 31 2020 for residents of the low SES vs high SES neighborhood. G) Relative risk of infection by Dec 31 2020 in the presence vs absence of evictions, for individuals who merged households due to evictions ("Doubled-up") and for individuals who kept their pre-epidemic household ("Other households"). Data in B), D)-G) shown as median values with interquartile ranges across simulations.

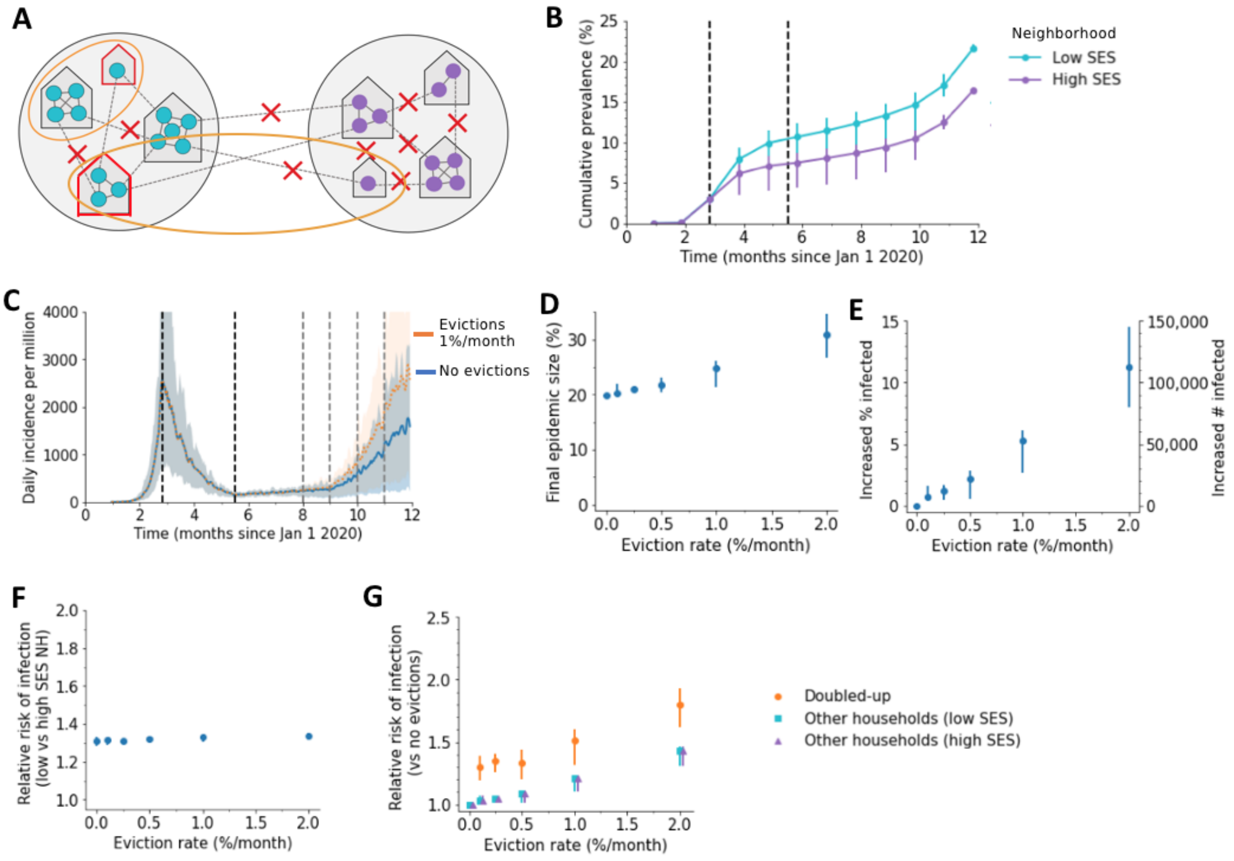

Supplementary Figure 15: Impact of evictions on COVID-19 epidemics in heterogeneous cities with cross-neighborhood doubling up. A) Schematic of our model for inequalities within a city. The city is divided into a "high socioeconomic status (SES)" (purple) and a "low SES" (teal) neighborhood. Evictions only occur in the low SES area (though doubling up is equally likely to happen with individuals in any area), and individuals living in the low SES area are assumed to be less able to adopt social distancing measures, and hence have higher contact rates under interventions (90% vs 80% reduction in external contacts during lockdown for 85% overall, 75% vs 65% during relaxation for 70% overall, and 65% vs 55% during fall comeback for 60% overall). Before interventions, residents are equally likely to contact someone outside the household who lives within vs outside their neighborhood. B) Cumulative percent of the population infected over time, by neighborhood, in the absence of evictions. C) The projected daily incidence of new infections (7-day running average) with 1%/month evictions vs no evictions. Shaded regions represent central 90% of all simulations. D) Final epidemic size by Dec 31 2020, measured as percent individuals who had ever been in any stage of infection, for the heterogenous city as compared to a homogenous city with same effective eviction rate and intervention efficacy. E) The predicted increase in infections due to evictions through Dec 31 2020, measured as excess percent of population infected (left Y-axis) or number of excess infections (right Y-axis). F) Relative risk of infection by Dec 31 2020 for residents of the low SES vs high SES neighborhood. G) Relative risk of infection by Dec 31 2020 in the presence vs absence of evictions, for individuals who merged households due to evictions ("Doubled-up") and for individuals who kept their pre-epidemic household ("Other households"). Data in B), D)-G) shown as median values with interquartile ranges across simulations.

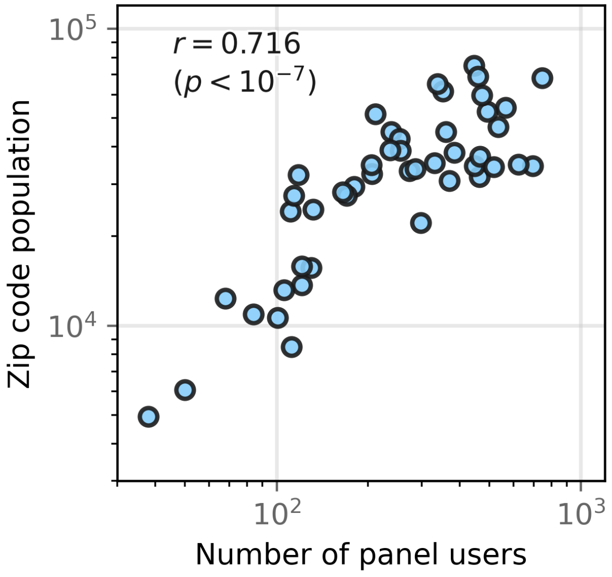

Supplementary Figure 16: Representativeness of the panel of Cuebiq users. For each zip code, we compare the number of sampled users in our panel with the zip code's total population. Pearson correlation was calculated on the log scale and the p value is for the null hypothesis of zero correlation.

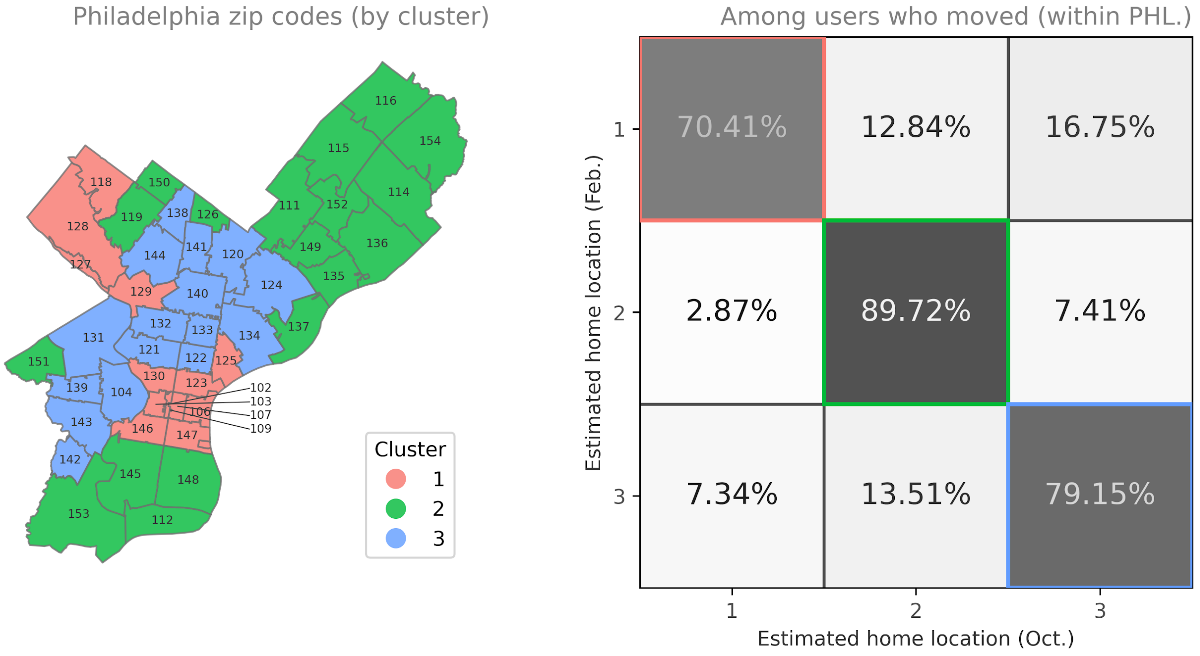

Supplementary Figure 17: Relocation probabilities between clusters. Left: Map of Philadelphia, with each zip code colored by the cluster it was assigned to. Right: Among users in our panel who have a different estimated home location between February and October 2020, this matrix shows the percent of users who relocated from/to each cluster. Note that most moves took place within the same cluster.

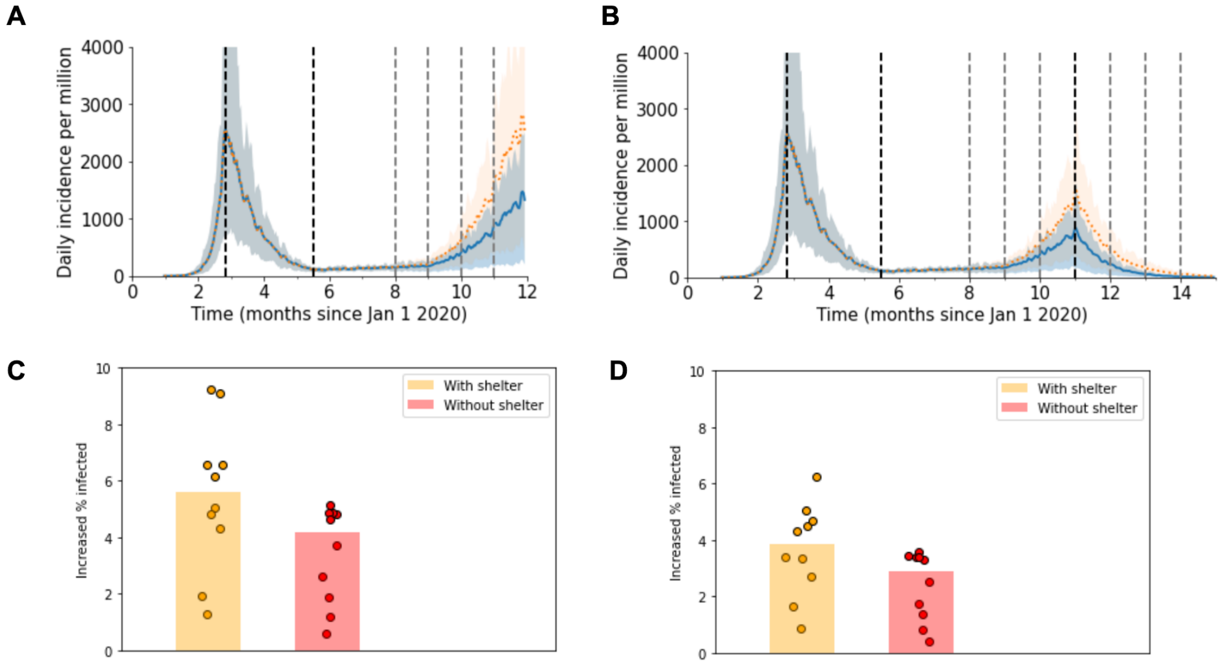

Supplementary Figure 18: Impact of evictions on a SARS-CoV-2 when evictions can lead to homelessness. We model evictions occurring in the context of an epidemic similar to cities following "Trajectory 1", with a large first wave and control in the spring, followed by relaxation to a plateau over the summer and an eventual comeback in the fall. Monthly evictions start Sept 1, with a 4-month backlog processed on the first month. Each month, 10% of evicted individuals become homeless and are assigned to a shelter, while the remaining 90% double-up. Each shelter size is drawn from a Poisson distribution with mean = 20 individuals. A)-B) The projected daily incidence of new infections (7-day running average) with (1% households per month) and without evictions. Shaded regions represent central 90% of all simulations.The first lockdown (dotted vertical line) reduced external contacts by 85%, under relaxation (second dotted line) they were reduced by 70%, and under the fall comeback they were reduced by 60% (fourth dotted line). In B) a second lockdown is imposed on Dec 1 and maintained through March 2021. C)-D) The predicted increase in infections through C) Dec 31, 2020 and D) March 31, 2021 due to evictions with (orange) and without (red) the inclusion of shelters for each of the above scenarios. It is measured in terms of an excess percent of the population infected. Bar plot shows the median predicted increase in infections.

Supplementary Tables

Supplementary Table 1: Parameters used for each simulated epidemic trajectory.

			Reduction in external contacts (%)				
	Base-line R_0	Cumulative prevalence at lockdown (%)	Early phase (pre March 25)	Lockdown (March 25)	Relaxation (June 15)	Control (July 15)	Comeback (Oct 1)
Trajectory Group (Figures 2, 3)							
1	3	3	0	85	70	70	60
2	3	1	0	85	65	75	60
3	3	0.2	0	77	65	80	60
4	3	0.2	0	75	60	80	65
Sensitivity analyses (Figures S10-S12)							
High SAR	3.5	3	0	87	75	75	65
Low SAR, Equal Weight	3	3	0	80	65	65	55
Equal Weight	3	3	0	85	70	70	60
Two-Neighborhood							
Low-SES	3	3	0	80	65	65	55
High-SES	3	3	0	90	75	75	65

Supplementary Table 2: Seroprevalence over time in simulated epidemic trajectories

	Seroprevalence (%)					
Scenario	April 1	June 1	September 1	December 1	January 1	March 1
Trajectory 1 & comeback	0.9 [0.5-1.0]	8.1 [4.9-9.1]	9.4 [6.0-10.5]	11.9 [9.1-12.8]	15.2 [13.9-16.4]	-
Trajectory 1 & lockdown	0.9 [0.5-1.0]	8.1 [4.9-9.1]	9.4 [6.0-10.5]	11.9 [9.1-12.8]	13.7 [11.8-14.6]	14.6 [13.1-15.6]
Trajectory 2 & comeback	0.3 [0.2-0.4]	3.4 [2.0-3.6]	4.4 [2.7-4.8]	6.1 [4.5-7.0]	9.2 [8.4-10.5]	-
Trajectory 2 & lockdown	0.3 [0.2-0.4]	3.4 [2.0-3.6]	4.4 [2.7-4.8]	6.1 [4.5-7.0]	7.7 [6.6-8.9]	8.7 [7.8-10.0]
Trajectory 3 & comeback	0.1 [0.0-0.1]	1.1 [0.7-1.1]	2.7 [1.9-2.8]	4.3 [3.2-4.6]	7.1 [5.8-7.9]	-
Trajectory 3 & lockdown	0.1 [0.0-0.1]	1.1 [0.7-1.1]	2.7 [1.9-2.8]	4.3 [3.2-4.6]	5.7 [4.6-6.4]	6.6 [5.5-7.4]
Trajectory 4 & comeback	0.1 [0.0-0.1]	1.2 [0.8-1.2]	4.3 [3.1-4.4]	6.5 [5.0-6.9]	8.4 [7.2-9.2]	-
Trajectory 4 & lockdown	0.1 [0.0-0.1]	1.2 [0.8-1.2]	4.3 [3.1-4.4]	6.5 [5.0-6.9]	7.6 [6.3-8.3]	8.3 [7.0-9.0]

Supplementary Table 3: Socioeconomic indicators used to classify zipcodes in Philadelphia into clusters.

		Mean by Cluster		
Indicator Description	Overall mean	Cluster 1	Cluster 2	Cluster 3
Population under 18 (%)	19.8%	11.4%	21.6%	24.5%
Population over 65 (%)	13.8%	13.7%	15.9%	11.4%
Female (%)	52.4%	51.1%	52.4%	53.5%
Poverty Rate (%)	22.9%	14.4%	17.6%	36.1%
Per Capita Income ($)	31,436	54,996	27,250	17,265
Median Household Income ($)	50,391	75,828	50,414	29,695
Median Home Value ($)	198,396	353,577	177,100	97,600
Renter Occupied (%)	47.6%	55.7%	37.5%	52.9%
Vacancy Rate (%)	12.6%	11.9%	9.2%	17.0%
Median Gross Rent ($)	1,092	1,448	1,023	885
Housing Cost Burdened Owners (%)	27.9%	25.1%	27.4%	30.8%
Housing Cost Burdened Renters (%)	48.9%	38.3%	50.8%	55.4%
Female Headed Households (%)	18.8%	7.7%	18.5%	28.1%
Residents Employed in Service Occupations (%)	22.2%	11.8%	23.2%	29.4%
Residents Employed in Essential Services (%)	46.2%	30.4%	50.4%	54.1%
Mobility Rate (% moved within last year)	15.9%	24.7%	10.5%	15.1%
Commute over 60 minutes (%)	14.4%	9.6%	15.1%	17.6%
Population (Thousands)	1,584	269	602	713
Proportion of Population (%)	100%	17%	38%	45%

Supplementary Table 4: Composition of zipcode-clusters in Philadelphia by race/ethnicity/nativity

	Cluster 1	Cluster 2	Cluster 3
White (non-Hispanic)	69.8%	51.1%	19.7%
Black	16.1%	34.1%	65.2%
Pacific Islander/American Indian/Alaska Native	0.2%	0.3%	0.4%
Asian	8.8%	7.8%	4.2%
Non-white	30.2%	48.9%	80.3%
Latino/Hispanic	6.6%	10.2%	17.7%
Foreign Born	11.5%	16.7%	10.1%

Supplementary Table 5: Baseline contact matrix by cluster of residence. Each row gives the fraction of all within-Philadelphia contacts involving individuals of a particular cluster that are with individuals residing in each other cluster. Contacts are estimated from co-location data from mobile devices (see Methods/Supplementary Note). These mixing patterns were calculated for January and February 2020 and are used as the baseline patterns to which all COVID-19-driven reductions are applied.

	Cluster 1	Cluster 2	Cluster 3	Total
Cluster 1	49%	17%	34%	100%
Cluster 2	18%	51%	31%	100%
Cluster 3	10%	13%	76%	100%

Supplementary Table 6 : Reduction in contacts by clusters of residence and month. Each row gives the percent reduction in contacts between individuals belonging to different clusters as compared to the baseline contacts. Reductions are for the months of April (top line in each row) and June (bottom line in each row) and are estimated from co-location data from mobile devices (see Methods/Supplementary Note).

	Cluster 1	Cluster 2	Cluster 3	Net
Cluster 1	94%<br>85%	99%<br>91%	99%<br>94%	96%<br>89%
Cluster 2	98%<br>88%	83%<br>64%	90%<br>77%	88%<br>73%
Cluster 3	98%<br>92%	92%<br>79%	91%<br>82%	92%<br>83%

Supplementary Table 7: Representativeness of the panel of Cuebiq users. For each zip code, we compare the demographic indicator for the entire population (from the US Census) to the estimated value for the panel of Cuebiq users. The anonymous nature of the Cuebiq data does not allow us to know demographic properties of individual users. Instead, we estimate the value for the panel by weighting the number of users in each zip code by the indicator value for that zip code.

Indicator Description	(Cuebiq panel value)/(Census value)
Median Age (years)	1.02
Population under 18 (%)	0.98
Population over 65 (%)	1.06
Per Capita Income ($)	1.01
Employment Rate (%)	0.99
Renter Occupied (%)	0.94
Mobility Rate (% moved within last year)	0.98
Residents Employed in Service Occupations (%)	0.98
Poverty Rate (%)	0.93
Female (%)	1.03
White (%)	1.19
Black (%)	0.85
Hispanic (%)	0.90

Supplementary References

1. Bi, Q. *et al.* Epidemiology and transmission of COVID-19 in 391 cases and 1286 of their close contacts in Shenzhen, China: a retrospective cohort study. *Lancet Infect. Dis.* **0**, (2020).
2. Lauer, S. A. *et al.* The Incubation Period of Coronavirus Disease 2019 (COVID-19) From Publicly Reported Confirmed Cases: Estimation and Application. *Ann. Intern. Med.* **172**, 577 (2020).
3. Kerr, C. C. *et al.* Controlling COVID-19 via test-trace-quarantine. *medRxiv* 2020.07.15.20154765 (2020) doi:10.1101/2020.07.15.20154765.
4. Larremore, D. B. *et al.* Test sensitivity is secondary to frequency and turnaround time for COVID-19 screening. *Sci. Adv.* **7**, eabd5393 (2021).
5. Du, Z. *et al.* Serial Interval of COVID-19 among Publicly Reported Confirmed Cases - Volume 26, Number 6—June 2020 - Emerging Infectious Diseases journal - CDC. doi:10.3201/eid2606.200357.
6. Wölfel, R. *et al.* Virological assessment of hospitalized patients with COVID-2019. *Nature* **581**, 465–469 (2020).
7. He, X. *et al.* Temporal dynamics in viral shedding and transmissibility of COVID-19. *Nat. Med.* **26**, 672–675 (2020).
8. Kim, S. E. *et al.* Viral kinetics of SARS-CoV-2 in asymptomatic carriers and presymptomatic patients. *Int. J. Infect. Dis.* **95**, 441–443 (2020).
9. Ali, S. T. *et al.* Serial interval of SARS-CoV-2 was shortened over time by nonpharmaceutical interventions. *Science* (2020) doi:10.1126/science.abc9004.
10. Lavezzo, E. *et al.* Suppression of a SARS-CoV-2 outbreak in the Italian municipality of Vo'. *Nature* (2020) doi:10.1038/s41586-020-2488-1.
11. Sanche, S. *et al.* High Contagiousness and Rapid Spread of Severe Acute Respiratory Syndrome Coronavirus 2. *Emerg. Infect. Dis. J.* **26**, (2020).
12. Tindale, L. C. *et al.* Evidence for transmission of COVID-19 prior to symptom onset. *eLife* **9**, e57149 (2020).
13. Zhou, F. *et al.* Clinical course and risk factors for mortality of adult inpatients with COVID-19 in Wuhan, China: a retrospective cohort study. *The Lancet* **0**, (2020).
14. Yang, X. *et al.* Clinical course and outcomes of critically ill patients with SARS-CoV-2 pneumonia in Wuhan, China: a single-centered, retrospective, observational study. *Lancet Respir. Med.* **0**, (2020).
15. Nande, A., Adlam, B., Sheen, J., Levy, M. Z. & Hill, A. L. Dynamics of COVID-19 under social distancing measures are driven by transmission network structure. *PLOS Comput. Biol.* **17**, e1008684 (2021).
16. Wu, J. T. *et al.* Estimating clinical severity of COVID-19 from the transmission dynamics in Wuhan, China. *Nat. Med.* 1–5 (2020) doi:10.1038/s41591-020-0822-7.
17. Verity, R. *et al.* Estimates of the severity of coronavirus disease 2019: a model-based

analysis. *Lancet Infect. Dis.* **0**, (2020).
18. Hauser, A. *et al.* Estimation of SARS-CoV-2 mortality during the early stages of an epidemic: A modeling study in Hubei, China, and six regions in Europe. *PLOS Med.* **17**, e1003189 (2020).
19. Levin, A. T. *et al.* Assessing the age specificity of infection fatality rates for COVID-19: systematic review, meta-analysis, and public policy implications. *Eur. J. Epidemiol.* **35**, 1123–1138 (2020).
20. Meyerowitz-Katz, G. & Merone, L. A systematic review and meta-analysis of published research data on COVID-19 infection-fatality rates. *medRxiv* 2020.05.03.20089854 (2020) doi:10.1101/2020.05.03.20089854.
21. Yang, W. *et al.* Estimating the infection-fatality risk of SARS-CoV-2 in New York City during the spring 2020 pandemic wave: a model-based analysis. *Lancet Infect. Dis.* **0**, (2020).
22. Miller, J. C. Spread of infectious disease through clustered populations. *J. R. Soc. Interface* **6**, 1121–1134 (2009).
23. Moreno, Y., Pastor-Satorras, R. & Vespignani, A. Epidemic outbreaks in complex heterogeneous networks. *Eur. Phys. J. B - Condens. Matter Complex Syst.* **26**, 521–529 (2002).
24. Princeton University. The Eviction Lab. *Eviction Lab* https://evictionlab.org/.
25. Stout Risius Ross, LLC. Estimation of Households Experiencing Rental Shortfall and Potentially Facing Eviction and Needed Relief for Those Households. *Stout.com* https://app.powerbi.com/view?r=eyJrIjoiNzRhYjg2NzAtMGE1MC00NmNjLTllOTMtYjM2NjFmOTA4ZjMyIiwidCI6Ijc5MGJmNjk2LTE3NDYtNGE4OS1hZjI0LTc4ZGE5Y2RhZGE2MSIsImMiOjN9 (2020).
26. Layser, M. D., De Barbieri, E. W., Greenlee, A., Kaye, T. & Saito, B. G. *Mitigating Housing Instability During a Pandemic*. https://papers.ssrn.com/abstract=3613789 (2020) doi:10.2139/ssrn.3613789.
27. Owens, A. Neighborhoods on the Rise: A Typology of Neighborhoods Experiencing Socioeconomic Ascent. *City Community* **11**, 345–369 (2012).
28. Aleta, A. *et al.* Modelling the impact of testing, contact tracing and household quarantine on second waves of COVID-19. *Nat. Hum. Behav.* 1–8 (2020) doi:10.1038/s41562-020-0931-9.
29. Ray, E. L. *et al.* Ensemble Forecasts of Coronavirus Disease 2019 (COVID-19) in the U.S. *medRxiv* 2020.08.19.20177493 (2020) doi:10.1101/2020.08.19.20177493.
30. Woody, S. *et al.* Projections for first-wave COVID-19 deaths across the US using social-distancing measures derived from mobile phones. *medRxiv* 2020.04.16.20068163 (2020) doi:10.1101/2020.04.16.20068163.
31. Chang, S. *et al.* Mobility network models of COVID-19 explain inequities and inform reopening. *Nature* 1–8 (2020) doi:10.1038/s41586-020-2923-3.
32. Kraemer, M. U. G. *et al.* The effect of human mobility and control measures on the COVID-19 epidemic in China. *Science* (2020) doi:10.1126/science.abb4218.
33. Coven, J. & Gupta, A. Disparities in mobility responses to COVID-19. (2020).
34. Huang, X. *et al.* The characteristics of multi-source mobility datasets and how they reveal the luxury nature of social distancing in the U.S. during the COVID-19 pandemic. *Int. J.*

Digit. Earth **0**, 1–19 (2021).
35. Jay, J. *et al.* Neighbourhood income and physical distancing during the COVID-19 pandemic in the United States. *Nat. Hum. Behav.* 1–9 (2020) doi:10.1038/s41562-020-00998-2.
36. Klein, B. *et al.* Reshaping a nation: Mobility, commuting, and contact patterns during the COVID-19 outbreak. 17.
37. Yabe, T. *et al.* Non-compulsory measures sufficiently reduced human mobility in Tokyo during the COVID-19 epidemic. *Sci. Rep.* **10**, 18053 (2020).
38. Aleta, A. *et al.* Quantifying the importance and location of SARS-CoV-2 transmission events in large metropolitan areas. *medRxiv* 2020.12.15.20248273 (2020) doi:10.1101/2020.12.15.20248273.
39. Klein, B. *et al.* Assessing changes in commuting and individual mobility in major metropolitan areas in the United States during the COVID-19 outbreak. 29.
40. Geohash. *Wikipedia* (2020).
41. United States Census Bureau. America's Families and Living Arrangements: 2019. *The United States Census Bureau* https://www.census.gov/data/tables/2019/demo/families/cps-2019.html (2019).
42. Nande, A. *et al. alsnhll/COVID19EvictionSimulations: The effect of eviction moratoria on the transmission of SARS-CoV-2*. (Zenodo, 2021). doi:10.5281/ZENODO.4589277.
