# Supplementary material for: The effect of eviction moratoria on the transmission of SARS-CoV-2
Source: Nat Commun. 2021 Apr 15;12:2274. doi: 10.1038/s41467-021-22521-5 (PMC8050248; doi:10.1038/s41467-021-22521-5)
Supplement: Supplementary file 2 — Reporting Summary [file 41467_2021_22521_MOESM2_ESM.pdf]

nature research

Corresponding author(s): Alison Hill

Last updated by author(s): Mar 6, 2021

Reporting Summary

Nature Research wishes to improve the reproducibility of the work that we publish. This form provides structure for consistency and transparency in reporting. For further information on Nature Research policies, see our Editorial Policies and the Editorial Policy Checklist.

Statistics

For all statistical analyses, confirm that the following items are present in the figure legend, table legend, main text, or Methods section.

n/a	Confirmed	
x		The exact sample size (n) for each experimental group/condition, given as a discrete number and unit of measurement
x		A statement on whether measurements were taken from distinct samples or whether the same sample was measured repeatedly
x		The statistical test(s) used AND whether they are one- or two-sided<br>*Only common tests should be described solely by name; describe more complex techniques in the Methods section.*
x		A description of all covariates tested
x		A description of any assumptions or corrections, such as tests of normality and adjustment for multiple comparisons
	x	A full description of the statistical parameters including central tendency (e.g. means) or other basic estimates (e.g. regression coefficient) AND variation (e.g. standard deviation) or associated estimates of uncertainty (e.g. confidence intervals)
	x	For null hypothesis testing, the test statistic (e.g. F, t, r) with confidence intervals, effect sizes, degrees of freedom and P value noted<br>*Give P values as exact values whenever suitable.*
x		For Bayesian analysis, information on the choice of priors and Markov chain Monte Carlo settings
x		For hierarchical and complex designs, identification of the appropriate level for tests and full reporting of outcomes
x		Estimates of effect sizes (e.g. Cohen's d, Pearson's r), indicating how they were calculated

Our web collection on statistics for biologists contains articles on many of the points above.

Software and code

Policy information about availability of computer code

Data collection	No data was generated in this study
Data analysis	Simulations were written in Python notebooks and run on Google Collaboratory using Python v3.6.9. Code is available in a Github repository: https://github.com/alsnhll/COVID19EvictionSimulations. Case and death data were processed using R 3.5.1, and dynamic time warping analysis was done using package dtw version 1.22-3

For manuscripts utilizing custom algorithms or software that are central to the research but not yet described in published literature, software must be made available to editors and reviewers. We strongly encourage code deposition in a community repository (e.g. GitHub). See the Nature Research guidelines for submitting code & software for further information.

Data

Policy information about availability of data

All manuscripts must include a data availability statement. This statement should provide the following information, where applicable:

- Accession codes, unique identifiers, or web links for publicly available datasets
- A list of figures that have associated raw data
- A description of any restrictions on data availability

All COVID-19 case and death data used in this study were downloaded from the publically-available New York Times repository in Github: https://github.com/nytimes/covid-19-data

Data on the distribution of US household sizes and on socio-demographic indicators of zipcodes in Philadelphia was obtained from the 2019 United States Census: https://www.census.gov/data/tables/2019/demo/families/cps-2019.html

Mobility data used in this study is available from Cuebiq through their Data for Good program (https://www.cuebiq.com/about/data-for-good/). Restrictions apply to the availability of this data, which was used under license for the current study, and so are not publically available. All data aggregated at the level of zip-code clusters that was used in the models is presented in the Supplemental Tables. More detailed data is available from the authors upon reasonable request and permission from Cuebiq, and any researchers interested in working with raw data can apply for an independent license from Cuebiq. Aggregated mobility metrics at the national, state, and CBSA level are publically available at https://covid19.gleamproject.org/mobility

Field-specific reporting

Please select the one below that is the best fit for your research. If you are not sure, read the appropriate sections before making your selection.

[x] Life sciences   [ ] Behavioural & social sciences   [ ] Ecological, evolutionary & environmental sciences

For a reference copy of the document with all sections, see nature.com/documents/nr-reporting-summary-flat.pdf

Life sciences study design

All studies must disclose on these points even when the disclosure is negative.

Sample size	This is not an experimental study; no data were generated. For Cuebiq mobility data, out of ~5 million users across the US, a subset was chosen based on living in the geographic region of interest (Philadelphia), leading to 13 thousand users being used for mobility analyses.
Data exclusions	This is not an experimental study; no data were generated. For the Cuebiq mobility data, the national panel of users they choose for all analyses excludes individuals if they don't satisfy one of the following criteria: 1) were active in the dataset for at least 21 days during each month from January to June 2020, 2) their devices reported on average at least one location ping per hour, and 3) had an average device geolocation accuracy of less than 50 meters.
Replication	This is not an experimental study; no data were generated. The only data used were Cuebiq mobility data. This is not an experiment so replication is not relevant. All stochastic simulations were repeated 10 times for each parameter value to calculate medians.
Randomization	This is not an experimental study; no data were generated. The only data used were Cuebiq mobility data. No intervention was applied so randomization is not relevant.
Blinding	This is not an experimental study; no data were generated. The only data used were Cuebiq mobility data. No intervention was applied so randomization is not relevant.

Reporting for specific materials, systems and methods

We require information from authors about some types of materials, experimental systems and methods used in many studies. Here, indicate whether each material, system or method listed is relevant to your study. If you are not sure if a list item applies to your research, read the appropriate section before selecting a response.

Materials & experimental systems

n/a	Involved in the study	
[x]	[ ]	Antibodies
[x]	[ ]	Eukaryotic cell lines
[x]	[ ]	Palaeontology and archaeology
[x]	[ ]	Animals and other organisms
[x]	[ ]	Human research participants
[x]	[ ]	Clinical data
[x]	[ ]	Dual use research of concern

Methods

n/a	Involved in the study	
[x]	[ ]	ChIP-seq
[x]	[ ]	Flow cytometry
[x]	[ ]	MRI-based neuroimaging
